# Brucella liver abscess; imaging approach, differential diagnosis, and therapeutic management: a case report

**DOI:** 10.4076/1757-1626-2-7143

**Published:** 2009-08-25

**Authors:** Danai Chourmouzi, Glykeria Boulogianni, Maria Kalomenopoulou, Ioannis Kanellos, Antonios Drevelegas

**Affiliations:** 1 Diagnostic Radiology Department, Interbalcan Medical CenterThessaloniki, 55236Greece; 2 Diagnostic Radiology Department, General Rethymnon HospitalRethymno 74100Greece; 3 Fourth Surgical Department, Aristotle University of ThessalonikiMacedonia, 55236Greece; 4 Radiologic Department, Aristotle University of ThessalonikiMacedonia, 55236Greece

## Abstract

We report a new case of a brucellar liver abscess (brucelloma) in a young woman without previous remote brucellosis who presented with pronounced systemic and mild local symptoms. Brucelloma is the result of calcification of a granoulomatous reaction induced by persistent *Brucella* in macrophages. It represents a rare manifestation that follows previously undetected brucellosis. We describe the findings in plain radiograph, ultrasound, computed tomography, and magnetic resonance images. Together with the positive serology, imaging yielded important elements supporting the diagnosis. Modern radiological techniques also contributed to the final therapeutic management, preventing unnecessary laparotomy. Sequencing confirmed the definite diagnosis of *Brucella melitensis* as the causative factor.

## Introduction

Diffuse hepatic involvement is common during the course of human brucellosis, given the affinity of *Brucella* for the reticuloendothelial system [1,2]. However, suppuration and liver abscess formation (brucelloma) are very rare complications, which may even be the presentation form of a previously undetected acute brucellosis [3].

To our knowledge, a case-based integrated imaging approach to both the differential diagnosis and therapeutic management of hepatic brucelloma has not yet been reported. We report a new case of a hepatic brucelloma in addition to 42 cases already existing in the medical literature [4], and we present the contribution of imaging techniques to the differential diagnosis and therapeutic management of hepatic brucelloma.

## Case presentation

A 34-year-old Greek woman was admitted to our hospital with a 2-month standing evening fever of up to 38.5°C, night sweats, arthralgia, and weakness. The patient had previously been in good health and had no prior episodes of suspected or diagnosed brucellosis. However, the patient was from a rural environment where brucellosis had been endemic until the early 1980s.

Physical examination revealed a normal blood pressure and heart rate, mild skin pallor, and mild systolic bruit of the mitral and aortic valves. The respiratory neurologic and musculoskeletal systems were found to be normal. No adenopathies were found on palpation except for a left submental, palpable lymph node, which was slightly tender. The abdominal physical examination revealed mild tenderness of the right hypochondrium with no peritoneal signs and a slight, smooth-edged right liver lobe hepatomegaly.

From the laboratory standpoint, the most significant disturbances included increased ESR (95 mm/h), normocytic normochromic anemia (Ht 31.5% and Hb 10 g/dL), a moderate hyper-leucocytosis (up to 11,100/mm^3^), and a slightly neutrophilic differential count. Liver function tests were normal and serology tests for hydatidosis, amebiasis, syphilis, and cytomegalovirus were negative. Circulatory antimitochondrial, antinuclear, and anti-smooth muscle antibodies were also negative; alpha-fetoprotein, carcinoembryonic and carbohydrate antigen 19-9 concentrations were normal. Serum agglutination tests for the detection of *Brucella* were positive at 1:160 and 1:128, while Coombs’ test for *Brucella melitensis* was positive at 1:640. Polyclonal IgG hypergammaglobulinemia was also noted, with IgA and IgM polyclonal antibodies within the normal range.

**Figure 1.**
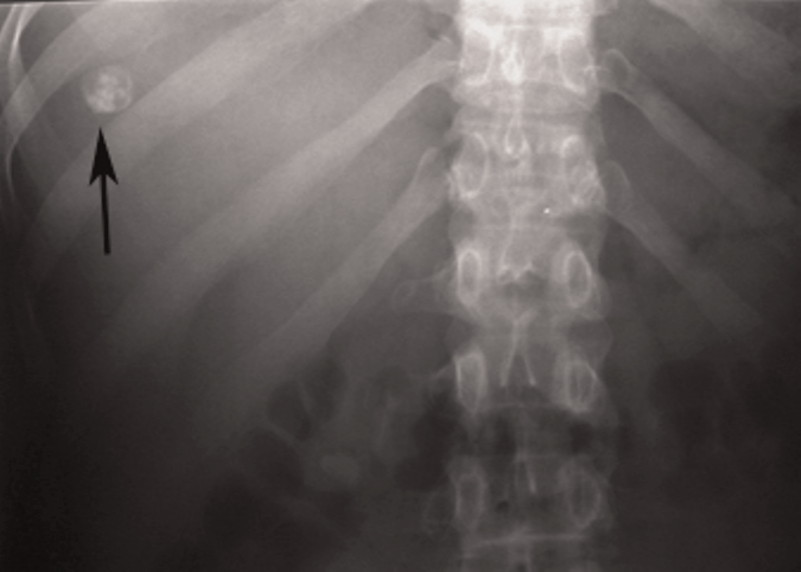
Plain film of the right hypochondrium shows a well defined, rounded calcification with an inner snowflake pattern.

**Figure 2.**
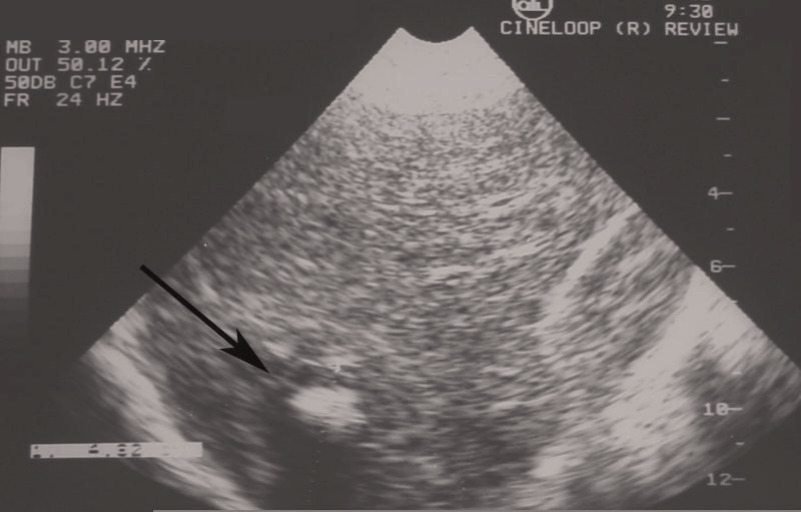
Ultrasound of the liver reveals a central hyperechoic lesion surrounded by a hypoechoic halo with ill-defined borders.

**Figure 3.**
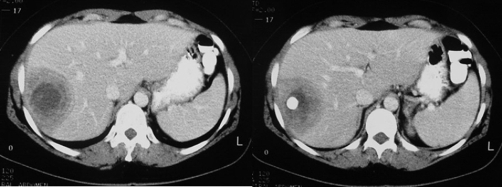
Contrast-enhanced CT scan reveals a hypoattenuating lesion in the right hepatic lobe with peripheral enhanced thickened wall. A central dense calcium deposit is also observed.

**Figure 4.**
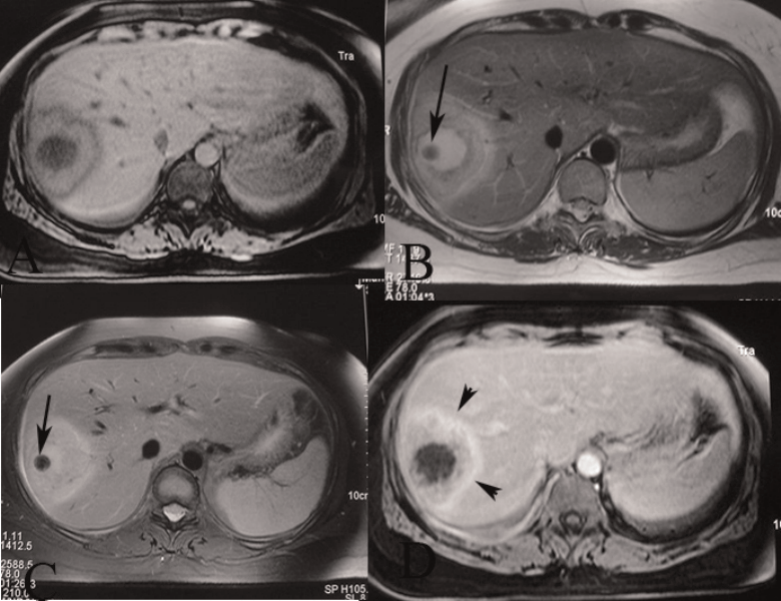
Axial MR images reveal **(a)** a central hypo-intense lesion on vibe, and hyperintensity on **(b)** T2-weighted and **(c)** STIR, with enchanced halo on gadolinium-enhanced images. **(d)** Periabscess liver tissue is hypointense on T1-weighted and mildly hyperintense on T2-weighted images, and reveals increased enhancement on post-gadolinium images (arrowheads). Note the dense calcium deposit as a low signal intensity lesion (arrows).

Plain radiograph of the abdomen revealed a rounded calcification with an inner snowflake pattern in the right hypochondrium (Figure 1). An abdominal ultrasound (US) revealed a dense central calcification surrounded by a solid hypoechoic halo with ill-defined borders in the dome of the liver (Figure 2); the remaining parenchyma was normal. Computed tomography (CT) scans and magnetic resonance imaging (MRI) confirmed the US findings, and also revealed further inner features of the mass-like liver lesion, including the nature of the lesion’s contours and changes in the surrounding liver parenchyma. A dense calcium deposit surrounded by a large heterogenous hypodense area was observed on CT scans (Figure 3), while in MRI with gadolinium contrast, the contours of the hypo-intense lesion in T1-weighted images appeared highly enhanced, producing an areola of hypervascularized liver parenchyma (Figure 4).

**Figure 5.**
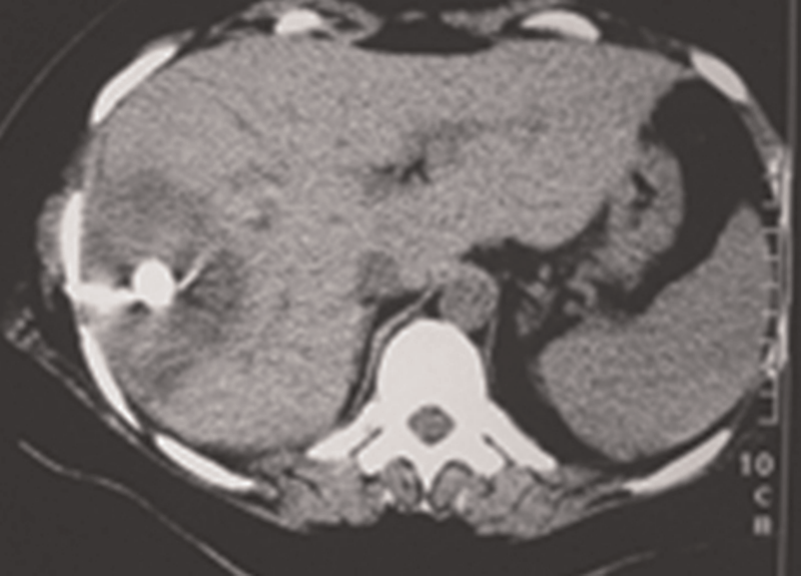
CT scan during percutaneous drainage under CT guidance reveals that the drainage catheter is in good position within the abscess cavity.

In view of the aforementioned clinical, serological, and radiographic findings, the diagnosis of a brucellar liver abscess was considered. Furthermore, bacteria was obtained from blood samples and cultured, and bacterial DNA was extracted and subjected to polymerase chain reaction (PCR). The PCR product was sequenced and found to be similar to *Brucellar melitensis*. Thus, the patient was initially managed with conservative therapy using 3 combined antibiotics: rifampicin (600 mg/day), doxicillin (100 mg/12 hrs) and streptomycin (1 g/day). However, failure of early response was considered because of persistent biological inflammatory syndrome for 2 weeks after treatment initialization. We decided to employ percutaneous drainage under the guidance of CT, since it is less aggressive than open surgery (Figure 5). Despite the thickness of the caseiform pus, its evacuation was effective. The patient received concomitant antibiotic therapy and had a good early response without relapse during a 6-month follow up. The patient’s outcome following pus drainage and 2 months of antibiotic therapy was considered satisfactory because both early and late response were effective at both 6 months and one year follow-up.

## Discussion

Liver and splenic involvement in patients affected by brucellosis is usually asymptomatic, although it is almost constant and diffuse, due to the important role of these organs in defending against *Brucella* infections [1,2]. However, brucellar hepatic or splenic abscess (brucelloma) is a remarkably rare complication [3] resulting from the caseous necrosis of granulomatous tissue that is induced by persistent *Brucella* in macrophages [4,5]. The brucelloma occasionally manifests during the course of acute brucellosis [6-8]. It more commonly represents a chronic form of the disease that has remained latent (as in our case). Thus, the brucelloma was the first clinical manifestation of a previously undetected case of acute brucellosis, and an integral part of the chronic stage of the disease.

The initial approach for the patient who presented with a chronic subacute inflammatory syndrome was based on both the evaluation of serological data and the imaging findings from US, CT, and MRI. The normal liver function tests and the slightly elevated serological titers for *Brucella* were carefully correlated with the liver mass and the central calcification detected by US, CT, and MRI. The liver mass was evaluated and its snowflake pattern appearance in the plain radiograph was considered consistent with the negative serology for parasitosis. Parasitosis was also easily excluded during differential diagnosis, due to additional imaging findings and negative serology tests for hydatidosis and amebiasis.

The sonographic pattern observed in our case was similar to that described in previously reported cases of hepatic brucellomas with sonographic evaluation [9,10]. This pattern of a solid, hypoechoic, centrally calcified lesion can consequently be considered as typical of brucellar liver abscess.

The CT evaluation provided further information about the exact location and extension of the lesion. This information proved very useful in treatment planning. The CT findings of liver brucellomas reported so far in medical literature are mostly of a rounded or ovoid hypodense area with central calcification, similar to our case [10,11,12].

To our knowledge, there are only 3 reports in the literature dealing with the MRI findings of hepatic brucelloma. They all agree with the typical pattern of a liver abscess, which is hypointense on T1 images and hyperintense on T2 images, as in our case. A peripheral tissue component, strongly enhanced after the injection of gadolinium contrast, as was also observed in our case, has been pointed out by Sisteron et al [4]. The combination of this feature with central calcification makes this imaging pattern atypical for an abscess, and can support the diagnosis of a brucelloma.

The serological behavior of chronic suppurative brucellosis may be misleading, because the tube agglutination testing titers are commonly borderline. Therefore, diagnosis must be based on both the modification of standard serological criteria, and correlation with the clinical and imaging findings. The anti-*Brucella* Coombs’ test with a titer of 1:640 was especially useful and is considered the best-suited test for detection of the serological response, which is expected to have the characteristics of a secondary reaction, with elevated concentrations of polyclonal IgG and normal IgM levels, as in our case. These data reflect a prolonged disease, the imaging features of which were compatible with long-standing liver involvement.

The definite diagnosis of brucellar liver abscess was accomplished by sequencing the PCR product from cultured bacterial DNA obtained from blood samples. It is well known that positive blood cultures, as well as cultures from percutaneously aspirated pus, are uncommon in prolonged cases. However, in our case a positive culture was detected between the second and third incubation week in a biphasic-modified medium. This allowed DNA extraction and PCR processing, which in turn gave positive results after sequencing for *Brucella melitensis*.

According to our case and the existing medical literature, interventional therapeutic management of the hepatic brucelloma is commonly recommended, since the patient’s outcome does not improve during conservative treatment. According to Ariza et al [13], late response can be considered as a cure since the patient remains free of brucellosis-related symptoms and no progression of radiological findings is observed.

In regions where brucellosis is endemic, brucelloma should be included in the differential diagnosis if a hepatic mass with central gross focal calcification is detected [14]. Imaging findings, together with a positive serologic test, confirm the diagnosis. Treatment should begin with rifampicin and doxycyclin. If medical treatment is unsuccessful, percutaneous drainage should be performed in order to prevent unnecessary laparotomy.

## Abbreviations

CT: computed tomography
MRI: magnetic resonance imaging
PCR: polymerase chain reaction
US: ultrasound
